# Freshwater Haplosporidian Diversity Associated With Invasive Non‐Native Bivalves in the River Thames, Including *Haplosporidium raabei* Infecting *Dreissena polymorpha*

**DOI:** 10.1111/jeu.70117

**Published:** 2026-09-01

**Authors:** Rachel Foster, Georgia M. Ward, Kelly S. Bateman, Paul F. Clark, Matthew J. Green, David Morritt, Paul Stebbing, David Bass

**Affiliations:** ^1^ Department of Life Sciences Natural History Museum London UK; ^2^ Centre for the Environment, Fisheries and Aquaculture Science Weymouth Laboratory Weymouth Dorset UK; ^3^ Department of Biological Sciences Royal Holloway, University of London Egham Surrey UK; ^4^ APEM Ltd. Manchester UK

**Keywords:** Haplosporida, invasive species, mussel, parasite, systematics

## Abstract

Haplosporidians are protistan parasites of aquatic invertebrates. Described here is the first report of the zebra mussel ( *Dreissena polymorpha* ) parasite *Haplosporidium raabei* in the River Thames, London, UK. The prevalence of this protist in the upper tidal Thames, Richmond, was higher than previously reported in other studies from the UK and mainland Europe of infected *D. polymorpha* populations, with molecular detection of *H. raabei* DNA in all *D. polymorpha* specimens collected. Also reported is the molecular detection (DNA and RNA) of *H. raabei* in other invasive Thames bivalves: the Asian clam ( *Corbicula fluminea* ) and quagga mussel ( *Dreissena bugensis* ). The molecular sequence of a closely related, but genetically distinct *H*. *raabei*‐like sequence associated with *D. polymorpha* is also reported. Two new PCR assays are presented for the specific and sensitive detection of *H. raabei* and *H. raabei*‐like sequences, enabling independent screening of these two lineages and also confirming the molecular presence of *H. raabei* in native unionid mussel species. A diversity of novel haplosporidian lineages associated with other invasive bivalves *C. fluminea* and *D. bugensis* and associated eDNA from the River Thames is also reported, demonstrating haplosporidian diversity associated with freshwater bivalves that is yet to be explored.

## 1 Introduction

The order Haplosporida (Ascetosporea) includes numerous parasites of marine mollusks and crustaceans, but the diversity of characterized freshwater haplosporidians remains limited (Hartikainen et al. 2014). Among the few described freshwater species, *Haplosporidium raabei* is a parasite of the freshwater bivalve *Dreissena polymorpha* (Molloy et al. 2012), *H. pickfordi* is known to infect the digestive gland of snails (Burreson 2001), and *H. diporeiae* is a parasite of amphipods described from the Laurentian Great Lakes (Winters and Faisal 2014). Environmental sequencing, however, has revealed a broader diversity than is currently reflected by described species, with numerous uncultured haplosporidians detected in freshwater lakes and sediments globally. This suggests that freshwater lineages in this group are more diverse and widespread than currently recognized (Ward et al. 2018).

Many haplosporidian lineages have been shown in close association with or as known parasites of marine bivalves (Lynch et al. 2020). This includes species implicated in mass bivalve mortality events such as *H. nelsoni* , *H. pinnae*, *H. edule*, *Minchina* spp. and WOAH‐listed *Bonamia ostreae* and *B. exitiosa* (Arzul and Carnegie 2015; Catanese et al. 2018). *Haplosporidium raabei* is at present the only described freshwater bivalve‐infecting haplosporidian and has been shown to cause lethal disease in the zebra mussel *D. polymorpha* , with pathology observed in the connective tissue of the gills, gonads, and digestive gland (Molloy et al. 2012). *Haplosporidium raabei* was originally identified within the Rhine and Meuse River catchments, spanning France, Germany, and the Netherlands (Molloy et al. 2012). Since its description in 2012, there were no further studies or records reporting this protistan parasite until a study of archived histology material revealed the presence of *H. raabei* in *D. polymorpha* collected from Grafham Water, Cambridge, England and Cardiff Bay, Wales in 2012 and 2013 (Foster, Hunt, et al. 2025).

*Dreissena polymorpha* , native to the Ponto‐Caspian region, is considered one of the world's most successful invasive species and has spread extensively in Europe and North America (Karatayev et al. 2015). Although it was first recorded in England in 1824, in the last 25 years their numbers have rapidly increased in some areas, including the River Thames (Aldridge et al. 2004). More recently other invasive freshwater bivalve mollusks have established in the Thames; the Asian clam, *Corbicula fluminea* in 2004 (Elliott and Ermgassen 2008) and the quagga mussel, *Dreissena bugensis* in 2014 (Aldridge et al. 2014). All three of these invasive bivalves are present in the upper tidal River Thames at Richmond, London (Pecorelli 2017, 2021). This Thames region also supports native unionid mussel species; the duck mussel ( *Anodonta anatina* ), painter's mussel ( *Unio pictorum* ) and the swollen river mussel ( *U. tumidus* ) (Ollard and Aldridge 2023).

Freshwater unionid mussels are one of the most endangered animal groups worldwide (Lopes‐Lima et al. 2018), primarily due to freshwater habitat degradation and the introduction of invasive non‐native mollusks (Carella et al. 2016). In particular, numbers of native unionid mussels in the Thames have declined significantly since the 1960s with their current estimated annual biomass production at just 7.5% of the level recorded in 1964 (Ollard and Aldridge 2023). The recent rise in the global spread of invasive freshwater bivalves presents a risk of potential parasite spillover of existing and novel parasites to threatened and declining populations of unionid mussels (Brian and Aldridge 2019). Invasive bivalve species may act as reservoirs or amplifying hosts for parasites within invaded ecosystems (Foster et al. 2021).

This study investigated co‐habiting native and invasive bivalve species present in the River Thames for haplosporidian parasites. We identify the presence of *H. raabei* molecular signal (DNA and RNA) in *D. polymorpha* tissue from the River Thames and provide the first evidence based on molecular signal to suggest it may be capable of infecting other freshwater bivalve species. We present a targeted PCR screen which shows more sensitive detection of the parasite in comparison to a “general haplosporidian PCR” (Hartikainen et al. 2014). We also describe the presence of a novel, closely related *H. raabei*‐like sequence type associated with *D. polymorpha* , and identify further diversity of novel haplosporidian lineages amplified from invasive bivalve tissue and eDNA from the River Thames.

## 2 Materials and Methods

### 2.1 Sampling

Sampling took place in the River Thames in November 2020 and November 2021 during the Port of London Authority Teddington Lock draw off at Richmond, London which allowed the riverbed to be accessed. Permission to sample was granted by the Port of London Authority. In November 2020, the riverbed was only accessible at one site due to high water levels from rainfall: Water Lane (51°27′32″ N, 000°18′30″ W). In November 2021, two sites were sampled; Water Lane and an additional site 1.2 km upstream, River Lane (51°26′55″ N, 000°18′21″ W). Bivalves were transported to Cefas laboratories, Weymouth, UK, in cool boxes lined with damp newspaper to maintain viability until dissection the following day. The number and type of bivalve species collected across both years combined are detailed in the results tables.

In both years, a large surface sediment sample (approx. 100 g) was taken from the exposed riverbed in the immediate vicinity of the bivalve collection site and preserved in molecular grade ethanol. In 2021 only, water samples were collected from the shoreline. Approximately 10 replicates of 120 mL water from both sites were manually filtered through 23 mm GF/F membranes (Whatman) using sterile syringes and filter holders (Sartorius, Germany). Dried filters were removed from the filter holder and preserved in 100% molecular grade ethanol. A further bulk water sample (6 L) was also collected from Water Lane in 2021 and stored in a cool box protected from light while in transport to the Cefas laboratories, where it was filtered under pressure onto 142 mm, 0.45 μm cellulose acetate filters (Sartorius, Germany). The filtrand was scraped from the filter membrane and divided into 2 subsamples, which were subsequently preserved in 2 mL cryotubes containing molecular grade ethanol at −20°C.

### 2.2 Dissection

To minimize the risk of cross‐contamination, each bivalve was dissected in a separate sterile Petri dish, and dissection blades and forceps were decontaminated between specimens using Virkon followed by ethanol. Bivalve shells were opened by severing the adductor muscle and a transverse section including mantle, gonad, digestive gland, gills, and foot was taken from the animal and fixed in Davidson's solution (Pioneer Research Chemicals Ltd., UK) for histological analysis. For DNA analysis, small sections of tissue were preserved in individual 2 mL tubes of RNALater (Invitrogen), with digestive gland tissue preserved and processed separately. Tissues were refrigerated overnight to allow permeation of RNALater into tissues before freezing at −20°C for longer term storage. DNA extractions were undertaken within 4 months, and RNA extractions were undertaken 2 years later. Small tissue sections were also preserved in glutaraldehyde for electron microscopy to be performed at a later date.

### 2.3 DNA Extraction and PCR

To perform the DNA extractions from bivalves, tissues were disrupted in 700 μL Lifton's buffer (100 mM EDTA, 25 mM Tris–HCl, 1% [v/v] SDS, pH 7.6) using Lysis Matrix A (MP Biomedical, USA) in a FastPrep‐24 instrument (MP Biomedicals for 1 min at 6 m/s). Following homogenization, 20 μL of 20 mg/mL Proteinase K (Sigma Aldrich, USA) was added and incubated overnight at 56°C. DNA was extracted from 100 μL of lysate per sample using the Maxwell RSC Tissue Kit (Promega, USA) on a Maxwell Rapid Sample Concentrator instrument (Promega, USA) as per the manufacturer's recommended protocol. DNA was eluted with 75 μL elution buffer and quantified using the QuantiFluor ONE dsDNA system (Promega, USA) using a Quantus fluorometer (Promega, USA).

To perform DNA extractions from sediment and water filters, preserved samples were freeze dried. DNA was extracted using the Qiagen Powersoil kit and quantified as above. Average (mean) DNA extraction concentrations were as follows: bivalve tissue slice 336 ng/μL; bivalve digestive gland 239 ng/μL; water eDNA 0.72 ng/μL; sediment eDNA 25 ng/μL. For PCRs, tissue and digestive gland extractions were diluted 1:50 with TE buffer.

PCR primers and cycling conditions for all PCRs used in this study are in Table 1. The “general haplosporidian” PCR assay used a nested PCR protocol, where 1 μL of product from the first reaction was used for the second round PCR. All haplosporidian PCRs targeted the 18S SSU (nuclear small subunit ribosomal RNA gene) region. Universal invertebrate COI primers from Folmer et al. (1994) were used to confirm species identification of the collected bivalves. All PCRs unless otherwise stated were conducted in 20 μL final volumes with 1 μL of template DNA and a final concentration of 0.5 μM of each primer, 0.4 mM dNTPs, 2.5 mM of MgCl_2_, 1× Promega Green GoTaq Flexi Reaction Buffer and 0.5 U of Promega GoTaq G2 Flexi DNA Polymerase. The full 18S SSU PCRs (~1600 bp) were conducted using a high‐fidelity polymerase: NEBNext High‐Fidelity 2X PCR Master Mix (New England Biolabs) in 25 μL final volumes with 1 μL of template DNA and a final concentration of 0.5 μM of each primer. Negative controls were also run for each PCR assay, comprising of extraction blanks from each run of the Maxwell RSC instrument and no‐template controls, in which the DNA template was replaced with the equivalent volume of molecular‐grade water.

**TABLE 1:** PCR primers and conditions used in this study.

Target	Primer set (5′–3′)	Expected product size (bp)	Cycling conditions	References
General Haplosporida nested (partial 18S)	Round 1	670	95°C 5 min, 30 × (95°C 30 s, 65°C 45 s, 72°C 1 min) 72°C 10 min (both rounds same)	Hartikainen et al. (2014)
	C5fHapl‐GTAGTCCCARCYATAAACBATGTC			
	Sb1n‐GATCCHTCYGCAGGTTCACCTACG			
	Round 2			
	V5fHapl‐GGACTCRGGGGGAAGTATGCT			
	Sb2nHap‐CCTTGTTACGACTTBTYCTTCCTC			
*H. raabei* specific screen (3′‐end 18S)	HR980f‐GTTGGACTTCCTCAGGACCCT	300	95°C 5 min 35 × (95°C 30 s, 63°C 45 s, 72°C 45 s) 72°C 7 min	This study
	HR1278R‐GCAAGCGGACGCTAGATAC			
*H. raabei*‐like specific screen (3′‐end 18S)	PR1190F‐CCAGGTCAAGACATAGTAAGGA	300	95°C 5 min 35 × (95°C 30 s, 63°C 45 s, 72°C 45 s) 72°C 7 min	This study
	PR1480R‐TTCTCAGGCCAGGCTGTG			
*H. raabei* complete 18S	HapGenF84_degen‐CTGTDAAACTGCAKATVGCTC	1600	95°C 5 min 37 × (95°C 1 min, 63°C 1 min, 72°C 2 min) 72°C 7 min	This study
	HR1278R‐GCAAGCGGACGCTAGATAC			
*H. raabei*‐like complete 18S	HapGenF84_degen‐CTGTDAAACTGCAKATVGCTC	1600	95°C 5 min 37 × (95°C 1 min, 63°C 1 min, 72°C 2 min) 72°C 7 min	This study
	PR1480R‐TTCTCAGGCCAGGCTGTG			
Universal Invertebrate COI	LCO1490‐GGTCAACAAATCATAAAGATATTGG	710	95°C 5 min 30 × (95°C 1 min, 50°C 1 min, 72°C 1 min) 72°C 7 min	Folmer et al. (1994)
	HC02198‐TAAACTTCAGGGTGACCAAAAAATCA			

*Note:* All PCR assays included an initial denaturation step at 95°C for 5 min, followed by the specified number of cycles of denaturation, annealing, and extension. Final extension conditions are indicated in the table.

Amplicons (2.5 μL) were visualized on 1.5% (w/v) agarose‐TAE gels stained with GelRed (Biotium, USA) and imaged using a UV transilluminator. Samples producing amplicons of the expected size were selected for downstream analysis and the remainder of these amplicons (17.5 μL) were purified using 0.8X reaction volumes of AMPure XP sample purification beads following the manufacturer's protocol (Beckman Coulter, USA). All PCR products were Sanger sequenced at NHM sequencing facility. Amplicons shorter than 600 bp were sequenced using the forward PCR primer from the corresponding PCR reaction only, while amplicons 600 bp or longer were sequenced using both forward and reverse primers to obtain overlapping sequence reads.

### 2.4 Cloning

General Haplosporida nested PCR amplicons from water and sediment samples were cloned to recover sequences from multiple Haplosporida lineages that may have been co‐amplified within a single PCR product. Amplicons were purified using AMPure XP sample purification beads as above, and an additional A‐tailing step was included to increase cloning reaction efficiency. They were A‐tailed by incubation at 72°C for 30 min in a reaction mixture of 50 μL final volume comprising 1X GoTaq colorless buffer, 2 mM MgCl_2_, 0.2 mM dATP, 5 U GoTaq (Promega), and 30 μL purified PCR product. A‐tailed amplicons were bead cleaned as above and ligated into pSC‐A‐amp/kan selective PCR cloning vectors (StrataClone, Agilent, USA) and transformed into heat‐shocked StrataClone SoloPack competent cells (Agilent). Cells were grown in 250 μL pre‐warmed Luria‐Bertani (LB) medium for at least 1 h at 37°C with agitation before plating onto LB‐ampicillin plates spread with 40 μL 2% X‐gal and incubated overnight at 37°C. Individual white colonies were picked using sterile 10 μL pipette tips and suspended into 20 μL PCR water. Suspended colonies were lysed at 95°C for 5 min in a thermal cycler, before the plasmid insert was amplified using M13 primers using the PCR reagent composition above and Sanger sequenced at the NHM.

### 2.5 RNA Extractions and cDNA Amplification

RNA was extracted from selected PCR‐positive individuals using the Zymo Quick‐RNA tissue/Insect kit following the manufacturer's protocol. PCR‐positive individuals were selected based on availability of remaining tissue and sufficient quantity and quality of the tissue on visual examination. RNA extractions were quantified using a Qubit 4 fluorometer using the RNA HS assay kit (Invitrogen). Average RNA extraction concentration was 38 ng/μL. RNA extractions were DNase treated using the Invitrogen DNA‐free DNA removal kit manufacturer's extended “rigorous protocol.” General invertebrate COI PCRs (Folmer et al. 1994) were conducted on treated RNA extractions to check for DNA contamination.

Single‐stranded cDNA was generated using the GoScript Reverse transcriptase kit (Promega, USA) following the manufacturer's protocol; using up to 5 μg RNA per reaction and equal parts Random Primers (0.5 μg/reaction) and Oligo(dT)_15_ (0.5 μg/reaction) and a final concentration of 3.75 mM MgCl_2_. Specific 18S *H. raabei* and *H. raabei*‐like PCRs were repeated on resultant cDNA as above; amplicon visualization, purification, and Sanger sequencing also followed as above.

### 2.6 Phylogenetic Analysis

Sequence chromatograms were edited in Geneious v2022.1.1 (Biomatters, New Zealand) to remove low quality bases and primer sequences before aligning against full SSU rRNA sequences for haplosporidia including representative sequences for all clades (including environmental‐only clades) using MAFFT v7 (Katoh et al. 2019). The alignment consisted of 80 sequences and 1836 nucleotide positions for almost full 18S coverage. No alignment masking was performed. Bayesian analyses were run using MrBayes v3.2.6 (Ronquist et al. 2012). Two separate MC3 runs with randomly generated starting trees were carried out for 5 million generations each with one cold and three heated chains. The evolutionary model included a GTR substitution matrix, a four‐category autocorrelated gamma correction, and the covarion model. 1.25 M generations were discarded as burn in. A Bayesian inference tree summarized by majority‐rule consensus was visualized in FigTree v1.4.4 and Bayesian Posterior Probabilities are indicated above 0.95.

**FIGURE 1:**
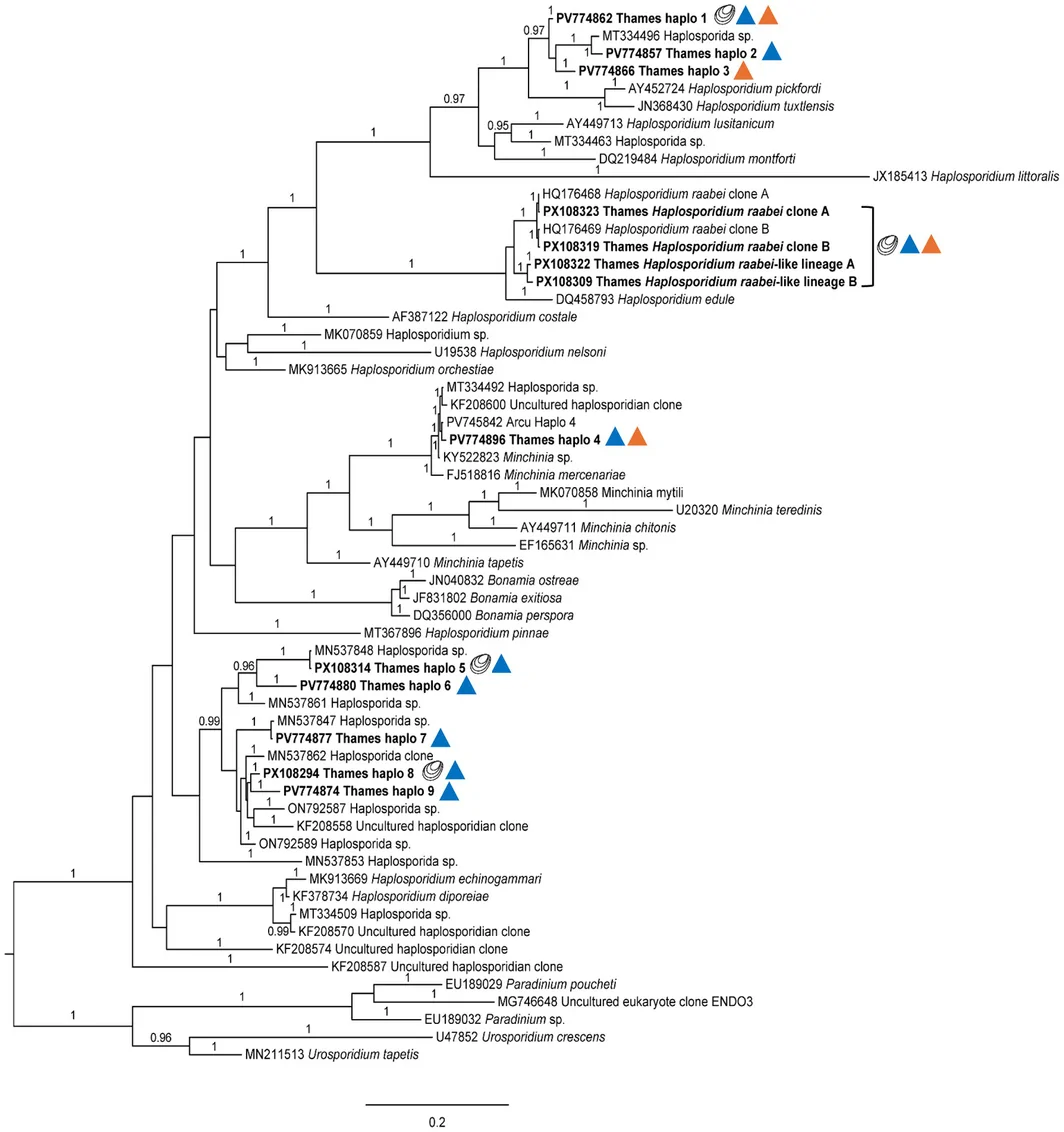
18S rRNA Bayesian tree of Haplosporida. Bayesian Posterior Probabilities are indicated on tree branches where above 0.95. Lineages amplified in this study are shown using a representative selection of sequences and are indicated on the tree in bold. A bivalve symbol denotes where a lineage was amplified from bivalve tissue. Blue triangles indicate amplification from water samples and orange triangles indicate amplification from sediment samples. The four sequences represented in the *raabei* clade were all found in bivalves, water, and sediment.

Environmental sequences generated in this study are deposited in GenBank under codes PV774856–PV774901 and sequences amplified from bivalve tissue are deposited under codes PX108294–PX108304 and PX108309–PX108324. Representative sequences were selected for the phylogeny in Figure 1; clades of diversity were collapsed if sequences had 6 or less nucleotide differences.

### 2.7 Histopathology

After a minimum of 48 h, Davidson's fixed samples were transferred to 70% industrial denatured alcohol. Samples were transferred to an automated vacuum infiltrating tissue processor (Peloris, Leica Microsystems, UK) for dehydration and paraffin wax infiltration. Paraffin embedded blocks were trimmed, and sections of 3–4 μm thickness cut using a rotary microtome (Finesse E/NE, ThermoFisher, UK) before mounting onto glass slides. Sections were stained using Hematoxylin and Eosin in an autostainer (Surgipath, UK), before examination under light microscopy for the presence of parasites and symbionts using a Nikon Eclipse Ni‐E (Nikon) light microscope.

## 3 Results

### 3.1 Molecular Screening of Bivalve and Environmental Samples

Using general, nested haplosporidian primers (Hartikainen et al. 2014), 18S sequences identical to *H. raabei* were amplified from 20 out of 306 bivalves from the Thames, from all three invasive species present (see Table 2). These nested primers also amplified a novel *H. raabei*‐like sequence with 97% similarity to *H. raabei* from 10 out of 306 bivalves. Individual *D. polymorpha* specimens were targeted to extend these two sequence types for nearly complete 18S coverage. A 1607 bp 18S sequence identical to HQ176468 *H. raabei* clone A was amplified, definitively confirming the presence of *H. raabei* in *D. polymorpha* from the Thames. The near‐full length 18S sequence of the *H. raabei*‐like lineage was also amplified giving a 1602 bp sequence, which had 95.97% similarity to both HQ176468 *H. raabei* clone A and HQ176469 *H. raabei* clone B. Across an overlapping 1598 bp region of the 18S SSU gene there are 59 nucleotide differences between *H. raabei* and *H. raabei*‐like sequences, which group sister to each other on the phylogeny in Figure 1.

**TABLE 2:** Incidence of *Haplosporidium raabei* and *H. raabei*‐like; from bivalves collected in 2020 and 2021 combined, amplified with general haplosporidian and targeted PCR.

Bivalve species	Total collected	*H. raabei* + (general PCR)		*H. raabei* + (specific PCR)		*H. raabei‐*like+ (general PCR)		*H. raabei*‐like + (specific PCR)	
		Tissue slice	Digestive gland	Tissue slice	Digestive gland	Tissue slice	Digestive gland	Tissue slice	Digestive gland
*Dreissena polymorpha*	12	5 (42%)	3 (25%)	12 (100%)	5 (42%)	5 (42%)	3 (25%)	10 (83%)	6 (50%)
*Dreissena bugensis*	44	4 (9%)	1 (2%)	14 (32%)	6 (14%)	0	0	3 (7%)	2 (5%)
*Corbicula fluminea*	213	11 (5%)	9 (4%)	159 (75%)	144 (68%)	0	5 (2%)	8 (4%)	24 (11%)
*Unio tumidus*	27	0	0	9 (33%)	9 (33%)	0	0	0	0
*Unio pictorum*	7	0	0	3 (43%)	4 (57%)	0	0	0	0
*Anodonta anatina*	3	0	0	1 (33%)	0	0	0	0	0

*Note:* All sequences were confirmed by Sanger sequencing.

Two specific PCR assays were designed (see Table 1) targeting a short 300 bp region at the 3′ end of the ribosomal 18S (SSU) of *H. raabei* and the *H. raabei*‐like lineage to maximize detection sensitivity and specificity. An increased frequency of molecular detection of these parasites in comparison to the general haplosporidian PCR was observed as shown in Table 2. Ten of the 12 *D. polymorpha* individuals collected were positive for both *H. raabei* and *H. raabei*‐like sequences using the targeted specific assays. Sanger sequencing confirmed there was no cross‐reactivity between the two PCR assays. Table 2 shows that the *H. raabei*‐like lineage was more restricted to *D. polymorpha* in comparison to *H. raabei*, which was found at a higher prevalence in *D. bugensis* and *C. fluminea* . None of the unionid mussels (native species) collected screened positive for haplosporidians using the general haplosporidian PCR assay, but positive PCR amplification was achieved for all unionid species when using the *H. raabei* targeted PCR. No unionid species screened positive for the *H. raabei*‐like targeted PCR.

A small subsection of samples were also extracted for RNA, further details of which are shown in Table S1. The targeted lineage specific PCRs for *H. raabei* and *H. raabei*‐like produced positive amplicons confirmed with Sanger sequencing from cDNA generated from DNA PCR‐positive individuals. *H. raabei* rRNA signal was detected in *D. polymorpha* , *D. bugensis* and *C. fluminea*, and *H. raabei*‐like rRNA signal was detected in *D. polymorpha* , and *C. fluminea* .

Other haplosporidian sequences were also amplified from bivalve tissue using the general haplosporidian primers, shown in the phylogeny in Figure 1. This included the presence of a prevalent novel haplosporidian lineage, which was found in 24/306 individuals sampled and showed an association with the non‐native species *D. bugensis* and *C. fluminea* . This lineage is referred to as Thames haplo 1, and the incidence of this lineage in bivalves is reported individually in Table 3.

**TABLE 3:** Incidence of novel haplosporidian lineage “Thames haplo 1” (amplified using general haplosporidian PCR).

Bivalve species	Total collected	Novel haplo 1 + (general PCR)		
		All + individuals	Tissue slice+	Digestive gland+
*Dreissena polymorpha*	12	0	—	—
*Dreissena bugensis*	44	12 (27%)	4 (9%)	9 (20%)
*Corbicula fluminea*	213	11 (5%)	8 (4%)	4 (2%)
*Unio tumidus*	27	1 (4%)	—	1 (4%)
*Unio pictorum*	7	0	—	—
*Anodonta anatina*	3	0	—	—

Environmental water and sediment samples taken from the Thames at the time of bivalve sampling also amplified a diversity of haplosporidian sequences, some of which were the same or highly similar to the diversity detected in the bivalve tissue which is shown in the phylogeny in Figure 1. Other amplified lineages showed similarity to previously detected environmental haplosporidians. The specific *H. raabei* and *H. raabei*‐like assays conducted on the eDNA samples showed molecular signals of these parasites were present in the sediment and water, but a higher incidence of *H. raabei* compared *to H. raabei*‐like (see Table 4).

**TABLE 4:** eDNA prevalence of *H. raabei* and *H. raabei*‐like using targeted PCR assays.

Year	Site	eDNA sample type	Replicates extracted	*H. raabei* PCR+	*H. raabei*‐like PCR+
2020	Water Lane	Sediment	3	3	1
2021	River Lane	Water (GF/F filter 120 mL)	9	8	1
2021	Water Lane	Sediment	3	2	1
		Water (GF/F filter 120 mL)	11	11	1
		Large volume water (6 L)	2	2	2

### 3.2 Histopathological Examination of Tissues

Histopathological examination was conducted on formalin‐fixed, paraffin‐embedded transverse sections comprising all major tissue types (including mantle, gill, digestive gland, and gonad) from all individual bivalves collected. For each specimen, a single section was examined in full for the presence of haplosporidian‐like cells, as well as other parasites and symbiotic organisms, using light microscopy. To aid the identification of haplosporidian cells, reference material for *H. raabei* comprising the type specimens obtained on loan from the Smithsonian Institution was examined, both by light microscopy and following high‐resolution digital slide scanning using Olympus VS200 Slideview digital slide scanner (as reported in Foster, Hunt, et al. 2025). This provided a comparative framework for recognizing characteristic developmental stages, including multinucleate plasmodia, sporocysts, and spores, within host tissues. Observations were recorded systematically during examination, as summarized in Table 5. No abnormality was detected in a large number of specimens examined. Microsporidian infection, *Morritospora corbicula*e (as described in Foster, Bateman, et al. 2025), was observed in the digestive gland tissues of *C. fluminea* and *D. polymorpha* . Single *U. tumidus* and *D. bugensis* specimens were noted to be infected with the microsporidian sampled in 2021. Across all specimens collected and examined, no cells consistent with *H. raabei* or other haplosporidians were observed.

**TABLE 5:** Summary of histological observations from all bivalve species sampled in 2020 and 2021.

Bivalve species	Sampling location	Sampling year	Number of animals	No anomaly detected (NAD)	*Morrittospora corbiculae* +
*Dreissena polymorpha*	Water Lane	2020	1	0	0
*Corbicula fluminea*	Water Lane	2020	147	124	23
*Unio tumidus*	Water Lane	2020	2	0	0
*Dreissena polymorpha*	River Lane	2021	7	3	4
*Dreissena bugensis*	River Lane	2021	23	23	0
*Corbicula fluminea*	River Lane	2021	15	11	4
*Unio tumidus*	River Lane	2021	22	22	0
*Unio pictorum*	River Lane	2021	7	7	0
*Anodonta anatina*	River Lane	2021	1	1	0
*Dreissena polymorpha*	Water Lane	2021	4	4	0
*Dreissena bugensis*	Water Lane	2021	21	20	1
*Corbicula fluminea*	Water Lane	2021	51	16	35
*Unio tumidus*	Water Lane	2021	3	2	1
*Anodonta anatina*	Water Lane	2021	2	2	0

Additional sections were examined from animals which showed a positive *H. raabei* signal via PCR analysis. Blocks were re‐cut, 100 μm of material was removed and then an additional section examined to determine whether infection could be observed elsewhere in the animal. No cells consistent with *H. raabei* or other haplosporidians were detected in any of the additional re‐cut sections.

The development of an in situ hybridisation (ISH) assay to enable the localisation of haplosporidian cells within host tissue was considered, using the mutually exclusive PCR assays designed to target *H. raabei* and the *raabei*‐like sequence type. However, sequence comparison shows that these lineages differ by only 10 nucleotide substitutions across the approximately 650 bp amplicon generated from the sampled material using the general haplosporidian (nested) primers, and these polymorphic sites were utilized in the design of the lineage‐specific primers. As a result, there is insufficient sequence divergence to identify regions suitable for the design of targeted, mutually exclusive ISH probes that would reliably discriminate between the two lineages. Given the high rate of co‐incidence of both sequence types within individual *D. polymorpha* hosts, a clade‐specific ISH probe would not be an appropriate approach for resolving lineage‐specific infections at the tissue level. Consequently, development of a lineage‐specific ISH assay was not feasible based on the available sequence data.

## 4 Discussion

### 4.1 Haplosporidum raabei

*Haplosporidum raabei* is currently the only known haplosporidian found to infect freshwater bivalves (Molloy et al. 2012). It is presumed to be rare (0.7% prevalence in mainland Europe and 0% in the USA) and previously measured prevalence rates within infected populations were < 4% (Molloy et al. 2012). A similarly low prevalence within infected populations was also reported from *H. raabei* detected in archival samples from Grafham Water and Cardiff Bay (Foster, Hunt, et al. 2025). In contrast, the findings of this study in the Thames suggest a much higher incidence of the parasite, with all the *D. polymorpha* specimens sampled testing positive for *H. raabei* using a targeted PCR assay (100% prevalence). It is important to note, however, the two previous studies were led by histological methods to screen tissues, and molecular methods were only used to confirm parasite identity (Molloy et al. 2012; Foster, Hunt, et al. 2025). This study has employed molecular‐led screening of bivalve tissues, which has resulted in substantially increased detection of the parasite compared to histological observations.

Using both the “general Haplosporida nested” assay and “specific *H. raabei*” assay, positives were more frequent in the tissue slice DNA compared to the digestive gland DNA. This is consistent with previous studies showing that *H. raabei* infections occur in connective tissues and are distributed across multiple organs (gill, gonad, digestive gland). Consequently, analyses based on broader tissue slices may increase detection probability compared with single‐organ sampling such as digestive gland alone.

This study also demonstrates a higher detection rate of *H. raabei* using the newly designed short‐amplicon assay than that achieved by the general haplosporidian assay, which shows the increased detection power of screening with species‐specific primers or a more efficient PCR. This has been demonstrated with other haplosporidians; a study using three PCRs of varying lengths to identify *H. pinnae* from water samples showed the PCR that generated the shortest amplicon was the most effective, possibly due to degradation of the DNA templates in water (Moro‐Martínez et al. 2023). Molecular biology theory also advises that short amplicons (less than 400 bp) are more easily amplified due a more efficient PCR reaction and lower possibility of secondary structures within the amplicon regions (Raymaekers et al. 2009).

Previous literature suggested that *D. polymorpha* is the only susceptible host of *H. raabei* (Molloy et al. 2012), but this study indicates other cohabiting bivalve species may host *H. raabei* as shown by our molecular detection protocol which amplified DNA and RNA from tissue samples. Although PCR prevalence was the highest in the known type host “ *D. polymorpha* ,” molecular sequences of *H. raabei* were derived from all the species collected. There was a lower incidence of *H. raabei* molecular signal in the three native unionid mussel species compared to the three invasive bivalve species ( *D. polymorpha* , *D. bugensis* and *C. fluminea* ; see Table 2), suggesting that the parasite is present at lower levels in the native species than the invasives. Haplosporidian host selection and partitioning has previously been observed among cohabiting marine bivalve species (Lynch et al. 2020).

Molloy et al. (2012) found two sequence variants of *H. raabei* with four base pairs difference, described as “clone A” and “clone B” (GenBank IDs: HQ176468, HQ176469). Both sequence variants were found to be present in the Thames. The *H. raabei* sequences were also amplified from filtered water and sediments taken at the time and location of the bivalve sampling. *Haplosporidium raabei* signal in associated eDNA water samples from the sampling sites indicates the presence of the parasite in the water column; however, this alone does not allow inference of planktonic hosts or dispersal stages. A wide diversity of other haplosporidians have been detected in water and sediment samples, including other bivalve pathogens such as *H. edule*, *Minchinia tapetis*, and *B. exitiosa* (Hartikainen et al. 2014), but specific life‐cycle stages of this group remain unresolved.

*Haplosporidium raabei* has previously been recorded in Western Europe, with infections observed upstream in the Meuse in France, as well as in the estuaries of the Meuse and Rhine in The Netherlands. It was theorized that the spread of *H. raabei* was facilitated by the freshwater estuary the rivers share in The Netherlands and the upriver connection where the Marne‐Rhine Canal links both basins (Molloy et al. 2012). Spatiotemporal data of freshwater invasions have shown an invasion corridor between the Rhine and Thames Estuaries, with the time lag between reports of new invasions in the Netherlands and Great Britian significantly decreasing over the last century (Gallardo and Aldridge 2015). Therefore, it is possible the source of *H. raabei* is from the movement of infected *D. polymorpha* from the Netherlands to the Thames. *Haplosporidium raabei* infections have also recently been detected in archival *D. polymorpha* samples collected in 2012 and 2013 from the UK; from Cardiff Bay and Grafham Water (Foster, Hunt, et al. 2025). These sites are not ecologically connected to the Thames catchment, but it is also possible infected *D. polymorpha* individuals could have arrived via contaminated boats or angling equipment transported from these sites. Considering the widespread distribution of *D. polymorpha* in the UK, it is also possible that *H. raabei* has always been more widely present and therefore further sampling of UK populations would clarify the distribution of the parasite. Furthermore, re‐sampling other sites across Europe with the improved molecular assay described in this manuscript may also reveal a more widespread distribution than previously reported by Molloy et al. (2012).

### 4.2 Histopathology Screening of Bivalve Tissues

Histopathology screening for the presence of haplosporidian‐like cells in all the sampled bivalve tissues was negative. The primary histopathological finding was evidence of infection with a microsporidian parasite observed to be highly prevalent in *C. fluminea* tissues and in a small number of other co‐habiting species and formally described as *Morrittospora corbiculae* in Foster, Bateman, et al. (2025).

Previous reports of histopathological detection of *H. raabei* in *D. polymorpha* populations have varied from 0.6% to 5% prevalence (with a single outlier of 20%) (Molloy et al. 2012; Foster, Hunt, et al. 2025). In the case of Molloy et al. (2012), when combining the data for the total of 5514 mussels examined using histological analysis, the overall infection prevalence was reported to be 0.7%. Only a total of 12 *D. polymorpha* individuals were collected in the dataset of 306 bivalves in this study, and therefore the statistical likelihood of observing an infection is low. Although an effort was made to collect an equal number of species, very few *D. polymorpha* specimens were located during sampling.

Molloy et al. (2012) state that there is a lack of evidence of seasonality of infection in *H. raabei*, with multiple developmental stages (including sporulation) observed in samples collected in the spring (April) through to the autumn (October). However, in their study limited sampling was conducted during the winter months, and so the occurrence and appearance of infection during the period spanning November through to March remains unclear. In the present study, all samples were collected in November, and therefore fall within this underrepresented window, providing additional context for interpreting the absence of histopathological detection of *H. raabei* in the sampled populations.

The two previous studies reporting the presence of *H. raabei* were led by histopathology, unlike this study which detected *H. raabei* using molecular methods. It is therefore possible *H. raabei* was more prevalent in the previously examined populations than could be determined via histology alone, or it may have gone undetected within other populations that were reported as histopathology negative (and therefore not followed up by molecular confirmation). Previous histopathological observations of *H. raabei* have primarily documented late‐stage sporogenesis (Molloy et al. 2012; Foster, Hunt, et al. 2025), suggesting that earlier developmental stages may be more difficult to detect and could account for the absence of histopathological evidence in the Thames population. Furthermore, if pathogenic effects are associated primarily with particular developmental stages, molecularly positive individuals may not necessarily exhibit observable pathology at all stages of infection.

Several haplosporidians have been shown to produce sub‐clinical infections, where molecular signal of the parasite is observed, but little or no evidence of pathology on histopathological examination (Arzul et al. 2024; Carnegie et al. 2000). For example, *H. nelsoni* in Pacific oysters *Crassostrea gigas* has been confirmed as being present with molecular signal and plasmodia present in heart smears, but with no haplosporidians seen in histology or in situ hybridization (Lynch et al. 2013). It has been suggested that histology does not become a reliable diagnostic tool for *H. nelsoni* until parasite density is approximately 10^3^–10^4^ parasites per g wet tissue (Ford 2010). Mass mortalities of Mediterranean mussels ( *Mytilus galloprovincialis* ) in the Adriatic Sea associated with a prevalent molecular signal of *Minchinia mytili* showed no evidence of the parasite upon histological examination (Zupičić et al. 2024).

In the present study, the development of an ISH assay was not feasible due to the limited sequence divergence available for probe design, particularly between the *H. raabei* and *raabei*‐like sequence types frequently co‐detected in bivalve tissues. Future work should therefore aim to generate additional sequence data from a greater number of specimens for both lineages, including full‐length ribosomal RNA sequences and alternative loci (e.g., actin genes), to facilitate the development of highly specific probes to improve the resolution of microscopic localisation of haplosporidian lineages within host tissues.

Despite the lack of observed infection in histopathology, RNA presence can provide evidence to indicate a viable parasite cell within the host (Finamore‐Araujo et al. 2022; Bass et al. 2023). Ribosomal RNA is abundant in living cells and therefore RNA‐based detection may provide a stronger indication of recent biological activity compared to DNA‐based detection (Blazewicz et al. 2013; Giroux et al. 2022). Furthermore, the lower environmental stability of eRNA compared with eDNA may reduce the risk of detecting residual nucleic acids originating from non‐viable organisms or environmental contamination (Kagzi et al. 2022). Following DNA extraction, a subset of samples were also extracted for RNA, and specific PCRs for *H. raabei* and *H. raabei*‐like sequences were conducted on generated cDNA. Only a small number of tissues were selected for extraction based on availability and visual examination of quality of leftover tissues; therefore, only *D. polymorpha* , *D. bugensis* , *C. fluminea* , and *Unio tumidus* specimens were available. The *H. raabei* cDNA PCR provided positive results from *D. polymorpha* , *D. bugensis* , and *C. fluminea* , and the *H. raabei*‐like assay gave positives from *D. polymorpha* and *C. fluminea*. The positive cDNA‐PCRs corresponded to individuals that were DNA‐PCR positive, and individuals that were DNA‐PCR negative were also cDNA‐PCR negative. The detection of rRNA in individuals that were also DNA positive may provide a more reliable indicator of viable cell presence in comparison to DNA signal alone and supports the interpretation that molecular detections reflect the presence of parasite cells within host tissues rather than extracellular or environmentally derived sequences. Furthermore, the detection of rRNA in additional bivalve species provides a basis for further investigation into whether the host range of *H. raabei* is restricted to *D. polymorpha* .

### 4.3 *Haplosporidum raabei*‐Like Sequences

*Haplosporidium raabei*‐like sequences were also detected within this population of Thames bivalves, grouping as sister to *H. raabei* on the Bayesian phylogeny in Figure 1. Similarly to clones A and B of *H. raabei*, there are 2 sequence variations of the *raabei*‐like sequence also shown in Figure 1.

In contrast to *H. raabei*, the *raabei*‐like sequences were predominantly amplified from *D. polymorpha* tissue (see Table 2), suggesting this sequence‐type is more restricted to this particular host. Similarly to *H. raabei*, the tissue slice DNA extractions provided more positives than the digestive gland samples, which may indicate a similar tissue trophism to *H. raabei*. Interestingly, the *raabei*‐like positives in *C. fluminea* were mainly restricted to the digestive gland samples, a pattern not observed for *H. raabei* in *C. fluminea* (see Table 2), which could indicate environmental acquisition via filter‐feeding processes rather than established infection.

Within *D. polymorpha* , all individuals tested positive for *H. raabei* using the lineage‐specific assay, and a subset of these same individuals also tested positive for the *H. raabei*‐like lineage using a separate lineage‐specific assay, demonstrating co‐detection of both lineages within individual hosts, providing direct evidence that both lineages can occur concurrently within the same host individuals in this system. RNA signal for the *H. raabei‐*like sequences was observed in *D. polymorpha* and *C. fluminea* providing further support for the presence of viable organisms originating from this lineage present within these hosts. As with *H. raabei*, no histopathological evidence attributable to the *raabei*‐like lineage was observed in infected hosts, and therefore its pathogenic significance remains unknown.

*Haplosporidium raabei*‐like sequences were not as prevalent in eDNA samples in comparison to *H. raabei* and were detected using the targeted PCR assay in 2/20 small volume water samples, 2/2 large volume water samples, and 2/6 sediment samples, in comparison to *H. raabei* which was detected in almost all of the eDNA samples (see Table 4). In addition, the *H. raabei*‐like sequences were not among the amplified haplosporidian clones in the haplosporidian‐wide cloned environmental libraries.

The differing pattern of host and environmental prevalence of the *H. raabei*‐like sequences in comparison to *H. raabei*, coupled with the phylogenetic evidence presented in Figure 1, suggests these sequences represent a separate novel species of freshwater bivalve‐infecting haplosporidian closely associated with *D. polymorpha* . In the absence of histopathology evidence, however, a full species description is not possible at this time.

### 4.4 Freshwater Haplosporidian Diversity

Several other freshwater haplosporidian sequences were amplified from invasive bivalve tissue and a diversity of haplosporidian sequences were recovered from eDNA water and sediment samples taken from the same sites. These have been grouped into nine novel lineages shown by representative sequences on the phylogeny (Figure 1). Three of these lineages (1, 5 and 8) were amplified from invasive bivalve tissue. Thames haplo 4 was highly abundant in both sediment and water eDNA and lineages 2, 6, 7 and 9 were amplified from water, demonstrating the diversity of haplosporidian signal from a freshwater environment.

Thames haplo 1 was the most prevalent lineage in the molecular data (excluding *H. raabei* and *H. raabei*‐like sequences) and was present in 2020 and 2021, amplified from *C. fluminea* , *U. tumidus* and *D. bugensis* tissues; therefore, its prevalence in each bivalve species has been individually reported in Table 3. This lineage was also detected frequently in eDNA water and sediment samples from the same sites. This lineage groups sister to *H. tuxtlensis* , described as a parasite of the striped false limpet, *Siphonaria pectinata* , from Mexico, and *H. pickfordi*, a parasite of freshwater snails (Burreson 2001; Vea and Siddall 2011). This lineage was detected in the highest association with *D. bugensis* tissue, with over a quarter of collected individual specimens amplifying this sequence (12/44—27%) in comparison to *C. fluminea* (11/213—5%). As this sequence was amplified with general PCR, detection could potentially be higher with a more specific targeted assay, as we have demonstrated to be the case for both *H. raabei* and *H. raabei*‐like.

Thames haplo 4 was amplified from numerous water and sediment samples and phylogenetic analysis placed this lineage within a well‐supported clade alongside *Minchinia mercenariae* and *M. mercenariae*‐like sequences. *Minchinia* spp. have primarily been detected from marine environments, and *M*. *mercenariae* is a parasite of marine bivalves, described from infections observed in hard clams ( *Mercenaria mercenaria* ) (Ford et al. 2009). *M. mercenariae*‐like sequences have been amplified from the European cockle ( *Cerastoderma edule* ) and the invasive Asian Date mussel (*Arcuatula senhousia*) (Ramilo et al. 2018; Foster, Dey, et al. 2025). The amplification of multiple sequences in this lineage from the Thames highly similar to previously detected *M. mercenariae*‐like sequences suggests either an unrecognized ecological plasticity within this lineage or the possibility of closely related freshwater‐specific *Minchinia* spp. It should also be noted that *Cerastoderma edule* is highly abundant and commercially fished in the Thames estuary (Tyler‐Walters 2007), therefore, this finding may also provide evidence that parasites of brackish hosts can be transported upstream in estuarine systems.

Thames haplo 5 was amplified from *C. fluminea* digestive gland DNA in two individuals and from multiple water samples. Sequences in this lineage were identical or highly similar to MN537848 isolated from the invasive Chinese mitten crab ( *Eriocheir sinensis* ) also collected from the Thames (Urrutia et al. 2019). Thames haplo 8 amplified from *D. bugensis* tissue and was almost identical to MN537862 isolated from tissue of the invasive red swamp crayfish ( *Procambarus clarkii* ) collected from a pond in Hampstead Heath, London (Urrutia et al. 2019). The recurrence of genetically similar haplosporidian sequences in other invasive hosts raises the possibility of cross‐species transmission or their environmental persistence within the Thames catchment. The repeated detection of these lineages in diverse invasive hosts highlights the role of biological invasions in promoting the establishment and spread of haplosporidian parasites in heavily invaded freshwater ecosystems such as the Thames.

## 5 Conclusion

This study revealed haplosporidian lineages associated with invasive bivalves and a novel diversity of lineages sampled from a freshwater environment. Documented here is the first molecular detection of *H. raabei* in multiple bivalve species, as well as a closely related but phylogenetically distinct sister lineage which requires further visual clarification for a species description to be made. The discrepancy between molecular detection and histological observations in this study is noteworthy and suggests that these haplosporidians may occur at low abundance, exhibit seasonal variation, or possess life‐cycle stages that are difficult to detect using conventional histopathology. The highly sensitive and specific *H. raabei* PCR assay presented in this manuscript could be used to re‐examine the known distribution of this parasite and investigate *H. raabei* pathology in other bivalve species, particularly in the context of threatened native unionid populations of conservation concern. Furthermore, this study has shown that highly invasive bivalve species *D. polymorpha* , *D. bugensis* , and *C. fluminea* could be a source and/or reservoir of haplosporidian parasites, which could represent potential risks to native bivalve species in invaded freshwater habitats as the ranges of current and future non‐native species expand.

## Funding

This work was supported by the Department for Environment, Food and Rural Affairs, UK Government (FB002) and Natural History Museum.

## Supporting information


**Table S1:** Lineage‐specific PCR detection of H. raabei and H. raabei‐like sequences from RNA (cDNA) extracts (green), with corresponding DNA‐based PCR results (blue) shown for comparison across the same host specimens.

## Data Availability

The data that support the findings of this study are uploaded in NCBI GenBank at https://www.ncbi.nlm.nih.gov/genbank/. These sequences have not been published yet, but will be released on publication of the article. Please contact r.foster@nhm.ac.uk if sequence data is required.
