# Supplementary material for: Freshwater Haplosporidian Diversity Associated With Invasive Non‐Native Bivalves in the River Thames, Including Haplosporidium raabei Infecting Dreissena polymorpha
Source: J Eukaryot Microbiol. 2026 Sep 1;73(5):e70117. doi: 10.1111/jeu.70117 (PMC13535272; doi:10.1111/jeu.70117)
Supplement: Supplementary file 1 [file JEU-73-e70117-s001.docx]

Supplementary Table 1.

| **Sample code** | **Species** | **Tissue Slice or Digestive gland** | **DNA *H. raabei +*** | **DNA *H. raabei*-like *+*** | **RNA *H. raabei +*** | **RNA *H. raabei*-like *+*** |
| --- | --- | --- | --- | --- | --- | --- |
| TB 150 | *C. fluminea* | DG |  |  |  |  |
| AC 43 | *C. fluminea* | DG | + |  |  |  |
| AC 43 | *C. fluminea* | DG | + |  |  |  |
| AC 44 | *C. fluminea* | DG | + | + | + |  |
| AC 44 | *C. fluminea* | TS | + | + |  | + |
| AC 95 | *C. fluminea* | TS | + |  |  |  |
| TB 150 | *C. fluminea* | TS | + |  |  |  |
| AC 43 | *C. fluminea* | TS | + |  |  |  |
| AC 150 | *D. polymorpha* | DG | + | + |  |  |
| TB 105 | *D. polymorpha* | DG | + | + | + |  |
| TB 10 | *D. polymorpha* | TS | + | + | + | + |
| TB 11 | *D. polymorpha* | TS | + | + |  | + |
| TB 102 | *D. polymorpha* | TS | + | + | + | + |
| TB 97 | *D. rostriformis* | TS | + |  | + |  |
| TB 50 | *U. pictorum* | DG | + |  |  |  |
| TB 54 | *U. timidus* | DG | + |  |  |  |
| TB 54 | *U. timidus* | TS | + |  |  |  |

Lineage-specific PCR detection of H. raabei and H. raabei-like sequences from RNA (cDNA) extracts (green), with corresponding DNA-based PCR results (blue) shown for comparison across the same host specimens.
